# Factors Influencing the Duration of Breastfeeding Among Polish Women

**DOI:** 10.34763/jmotherandchild.2020241.2006.000007

**Published:** 2020-07-29

**Authors:** Julia Tracz, Danuta Gajewska

**Affiliations:** 1 Department of Dietetics, Institute of Human Nutrition Sciences, Warsaw University of Life Science, Warsaw, Poland

**Keywords:** breastfeeding, breastfeeding termination, Polish mothers

## Abstract

**Objective:**

The study aimed to analyse the factors that influence the duration of breastfeeding among Polish women.

**Materials and methods:**

The study group consisted of 1,024 mothers of children aged 6–18 months who were breastfeeding or are currently breastfeeding. Data were collected through a computer-assisted Web interview. Univariate analysis and correspondence analysis were performed to determine the predictors of exclusive breast-feeding and breastfeeding among Polish women.

**Results:**

Maternal age, maternal education, pre-pregnancy body mass index, voivodeship, birth weight and due time had a significant impact on breastfeeding duration (p < 0.05). Mothers who were older (35± years of age), had a higher level of education and lived in mid-sized cities were more often breastfeeding exclusively, in accordance with the World Health Organization's recommendations. Women who were younger, had a lower level of education, lived in rural areas, and had a lower BMI breastfed exclusively for a shorter period. The most frequently suggested reason for breastfeeding cessation was maternal concerns about insufficient milk supply (41%). The percentage of women who gave up breastfeeding before the 6th month following the delivery was the highest in the northeastern region of Poland (53%), whereas the percentage of women who stopped exclusive breastfeeding was the highest in the southwest region of Poland (69.4%).

**Conclusions:**

Our study highlights that the reasons for breastfeeding cessation are often complex. Therefore, the promotion of breastfeeding for the first 6 months of life should be a social responsibility.

## Background

Breastfeeding is a gold standard for infants’ nutrition worldwide. The World Health Organization (WHO) recommends exclusive breastfeeding up to 6 months of age with continuous breastfeeding along with appropriate complementary foods up to 2 years of age or beyond (1). According to WHO, exclusive breastfeeding implies that the infant has received only breast milk (2).

Both mother and child benefit from breastfeeding. Concerning the woman's health, breastfeeding leads to a faster recovery after delivery by decreasing the risk of postpartum bleeding and accelerating involution of the uterus; a faster return to pre-pregnancy weight; the prevention of breast and ovarian cancers and a lower risk of postpartum depression (3,4,5,6,7). For the child, breastfeeding reduces the risk of gastrointestinal and respiratory system infections, sudden infant death syndrome (SIDS), sepsis, necrotizing enterocolitis, inflammatory bowel disease and obesity in adult life, and it may have a protective effect against allergic diseases (3,4,5,6, 8). Although there are clear recommendations that are related to the duration of breastfeeding and beneficial influence on mother's and child's health, the percentage of children who are breastfed is very low. Breastfeeding at 12 months ranges from <1% in the UK to 35% in Norway. In low-income and middle-income countries, only 37% of children aged under 6 months are exclusively breastfed (1). The available data from Poland indicate that the percentage of breastfeeding women is likewise low (9). In 2017, Królak-Olejnik et al. found a breastfeeding initiation rate of 97% and a 6-month exclusive breastfeeding rate of 4% (10).

The decision about breastfeeding is determined by various social and cultural factors. Therefore, improving the knowledge about the factors influencing breastfeeding and promoting the benefits of breastfeeding among women should be an overarching priority for the whole society. There is a strong need to evaluate the trends of breastfeeding duration and reasons for its cessation on a large population. Findings from this type of research may be helpful in implementing appropriate and effective actions to promote exclusive breastfeeding as a gold standard.

## Aim

This study aimed to analyse the factors that influence the duration of breastfeeding among Polish women.

## Materials and Methods

This is a nationwide survey, which included 1,024 mothers between 18 and 50 years old who were breastfeeding or are currently breastfeeding. Data were collected between April and May 2018 using a questionnaire. The study was a part of the educational programme for parents ‘*Healthy start to the future*’. The coordinator of this project was the Polish Society of Dietetics and the main partner was Nestlé. The detailed design and methods of the study have been described elsewhere (9).

Purposive sampling was used to select the study sample. The inclusion criteria of the study were being a breastfeeding woman (adult) and being a mother of an infant or toddler aged 6–18 months. The exclusion criteria of the study were being pregnant, being a woman who has never breastfed, being the mother of infants aged <6 months or toddlers aged over 18 months.

The study was conducted by using computer-assisted Web interview (CAWI), obtaining information from respondents who completed the individual electronic form on the panel destined for Internet users (Opin.pl) of the independent research agency IQS. The survey was anonymous. The time for completion of the survey was no longer than 15 min. The questionnaire included the questions about pregnancy, childbirth, breastfeeding and mother's dietary habits during pregnancy and breastfeeding, as well as demographic data. The study was conducted in line with the ethical principles specified in the Declaration of Helsinki of 1964.

All data were analysed using STATISTICA version 13 (StatSoft, Inc). In the descriptive characteristic of categorical variables, the number of observations and the percentage of occurrences were considered. The differences in demographic variables between groups were evaluated using a Chi-squared test. Univariate analysis and correspondence analysis were performed to determine the predictors of exclusive breastfeeding and breastfeeding among Polish women. The significance level for all statistical analyses was considered at *p* ≤ 0.05.

## Results

Detailed characteristics of the study group have been described in the recent study (9). Briefly, the majority of mothers were aged between 25 and 34 years (46%). More than half of them had higher education (52%). The percentage of mothers living in cities was 60%. Abnormal pre-pregnancy body mass index (BMI) was recorded for 30% of the participants. The percentage of women who delivered by caesarean section was about 40%, and more than half of the participants gave birth on term. As previously reported, 64% of the studied population were mothers who have breastfed in the past and 34% were mothers who are currently breastfeeding. Only 21% of women declared to have exclusively breastfed for 6 months (9).

**Figure 1:**
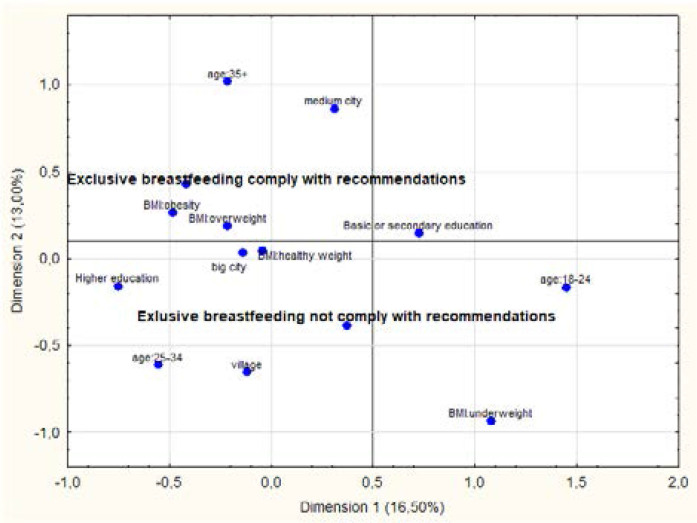
Characteristic of women meeting with WHO recommendations about duration of exclusive breastfeeding (Correspondence Analysis)

Table 1 and Figure 1 show breastfeeding duration according to the general characteristics of the study population. The largest percentage of women who gave up breastfeeding before 6 months after delivery was noted among women living in the rural area. There were significant differences in breastfeeding duration based on maternal age and the level of mother's education.

**Table 1:** The distribution of breastfeeding time depending on the socio-demographic factors of mothers and the type and time of delivery

**Characteristics**	**Breastfeeding mothers (N=1024)**	**Time of breastfeeding**					**p-value**

		**<1 month**	**1–2 months**	**3–5 months**	**6–8 months**	**≥9 months**	
**Maternal age** [%]							
18–24 years	227 (22%)	7.9	25.1	20.7	22.9	23.3	.000
25–34 years	466 (46%)	3.4	16.1	21.5	24.7	34.3	
35+ years	331 (32%)	2.7	7.9	19.6	25.7	44.1	
**Education** [%]							
Primary and secondary	495 (48%)	5.3	19.1	21.4	22.9	31.3	.001
Higher	529 (52%)	3.0	11.6	20.0	26.4	39.0	
**Place of living** [%]							
Village	412 (40%)	5.8	17.7	19.4	21.8	35.2	.102
Medium city	304 (30%)	4.3	14.1	19.1	27.0	35.5	
Big city	308 (30%)	1.9	13.6	24.0	26.0	34.4	
**Pre-pregnancy BMI (kg/m^2^)**[%]**							
Underweight	82 (8%)	4.9	24.4	22.0	17.1	31.7	.210
Healthy weight	693 (70%)	3.8	14.0	20.2	25.3	36.9	
Overweight and obesity	169 (17%)	5.3	16.0	23.7	28.40	26.6	
Obesity	47 (5%)	6.4	17.0	21.3	19.1	36.2	
**Mode of delivery** [%]							
Vaginal Birth	613 (60%)	3.3	13.7	21.2	25.9	35.9	.102
Caesarean section	411 (40%)	5.6	18.0	20.0	22.6	33.8	
**Due time** [%]							
Within due time	652 (64%)	4.0	15.0	21.6	25.2	34.2	.301
Premature <37 weeks	101 (10%)	5.9	21.8	21.8	23.8	26.7	
After the date	271 (26%)	4.1	14	18.1	23.6	40	

* Pearson's Chi-2 test, differences between groups;

** Number of responses less than 1024

Mothers who were older (35+ years of age), had a higher level of education and lived in mid-sized cities were more often breastfeeding exclusively, in accordance with WHO's recommendations. Women who were younger, had a lower level of education, lived in rural areas and had a lower BMI breastfed exclusively for a shorter period.

**Figure 2:**
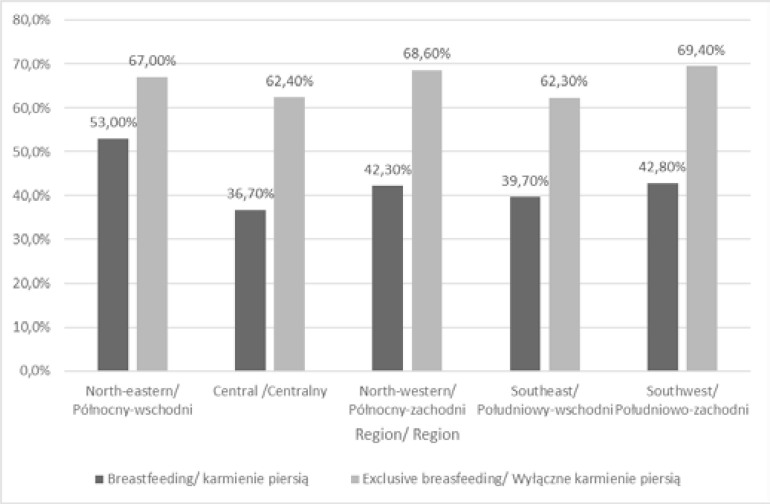
Percentage of women participating in the study who stopped breastfeeding or exclusive breastfeeding before child turned 6 months, by regions of Poland (Pearson's Chi-2 test, differences between groups)

The percentage of women who gave up breastfeeding before the 6th month following the delivery was the highest in the northeastern region of Poland (53%), whereas the percentage of women who stopped exclusive breastfeeding was the highest in the southwest region of Poland (69.4%). Most mothers who declared to have continued any form of breastfeeding for 6 months or longer lived in Central and Southeast of Poland. A significant difference in breastfeeding duration and geographical differentiation was observed only between breastfeeding groups (*p* < 0.05) (Figure 2).

Results from the univariate analysis are shown in Table 2. Maternal age and BMI were found to be the factors significantly influencing exclusive breastfeeding duration, whereas maternal age, mother's education level, voivodeship, birth weight and due time had a significant influence on breastfeeding duration in general.

**Table 2:** Factors influencing exclusive breastfeeding and breastfeeding among study group

**Variables**	**Exclusive Breastfeeding**	**Breastfeeding**

	**p-Value**	**p-Value**
Maternal age	**0,0000**	**0,0000**
Mother's education	0,1254	**0,0167**
Pre-pregnancy BMI	**0,0165**	0,0660
Place of living	0,0828	0,5505
Voivodeship	0,6532	**0,0494**
Mode of delivery	0,1636	0,3560
Birth weight	0,1254	**0,0282**
Due time	0,8917	**0,0332**

The most frequently suggested reason for breastfeeding cessation was lack of milk (41%). Among women declaring lack of milk, almost three-fourths gave up breastfeeding, and more than 85% ceased exclusive breastfeeding within 6 months after the delivery. More than half of the respondents gave up breastfeeding after 9 months following the delivery because of the age of their child, while half of the mothers gave up exclusive breastfeeding before 6 months after the delivery for the same reason. Difficulties in breastfeeding and insufficient milk supply were the reasons for giving up exclusive breastfeeding mainly in the first month after the child's birth (Table 3).

**Table 3:** Time of exclusive breastfeeding depending on the reasons for cessation of breastfeeding declared by women participating in the study

**Reasons for the cessation of exclusive breastfeeding declared by women participating in the study**	**Total** N=733*	**Exclusive breastfeeding duration (months)**					p-value**

		**<1 month**	**1–2 months**	**3–5 months**	**6–8 months**	**≥9 months**	
Insufficient milk supply	300 (41%)	25.3	29.6	32.6	11.7	0.9	<0.001
Difficulties in breastfeeding	101 (14%)	31.7	25.8	33.6	8.9	0.0	<0.001
Breastfeeding cessation because of child age	153 (21%)	0.0	5.3	44.4	35.3	15	<0.001
Feeding with modified milk was more convenient	90 (12%)	22.2	22.2	32.3	20.0	3.3	0.590
Other reasons	89 (12%)	14.6	15.8	43.9	19.0	6.7	0.615

* Number of responses

** Pearson's Chi-2 test, differences between groups

## Discussion

Our results are consistent with a negative tendency in the literature, which describes that actual breastfeeding duration does not meet the WHO recommendations (1, 10,11,12]. In developed countries, less than one out of five children are being breastfed up to 12 months of age (1). In this study, we found that maternal age, maternal education, pre-pregnancy BMI, voivodeship, birth weight and due time had a significant impact on breastfeeding duration.

Our findings indicate that young maternal age (18–24 years old) is negatively associated with breastfeeding duration corroborate with the previous result (13, 14). In the study by Ogbo et al., the younger age of the mother (<20 years old) was associated with faster termination of exclusive breastfeeding in the early postnatal period in comparison with women aged 20–34 years old (15). Wallenborn et al. highlighted that women whose pregnancy was unintended were less likely to breastfeed. Younger women (<19–24 years old) more often had unintended pregnancies (16). There is a strong need to highlight the importance of both sexual education for adolescents and the facilitation of access to modern contraceptive methods. Considering the age group and different needs related to age, there is an opportunity to create an accurate educational programme that could prolong the duration of breastfeeding.

BMI before pregnancy was one of the factors significantly affecting the duration of breastfeeding. Our finding indicates that both underweight and overweight women gave up breastfeeding before the 6th month after the child's birth, in 48 or 46% of cases, respectively, while the percentage of mothers with normal weight who weaned before the 6th month after delivery was 59% (9). In the study carried out among 3,033 women, it was found that obesity was associated with a greater risk of faster termination of exclusive breastfeeding (17). Obese women may have hormonal and metabolic disorders that lead to problems with breastfeeding. Moreover, children can have trouble with sucking due to the larger size of the mother's breast and nipples (18). Therefore, healthcare professionals are bound to ensure women who are maintaining a healthy weight is crucial not only to the health of the mother or child but that it also affects the course of lactation. Knowing the risk that abnormal BMI has on breastfeeding duration, healthcare professionals should undertake steps to maximize breastfeeding length in this group. These issues should be emphasized to women during pregnancy planning. In the Hauff et al. study, overweight and obese women were exposed to many psychosocial factors (such as social knowledge and maternal confidence in breastfeeding) that were related to faster weaning (17). Taking that into account, support from family, society and healthcare professionals should be provided.

In this study, the percentage of women who deliver by caesarean section was high (40%). However, we did not find any significant difference between the mode of delivery and the time of breastfeeding termination. The Organization for Economic Cooperation and Development (OECD) report noted the rapid growth of caesarean section rate in Poland compared with other European countries. In 2017, a caesarean section in Poland was one of the highest across OECD countries, with a rate reaching 39.3% of all live births (19). It is believed that unplanned and planned caesarean section may have an adverse effect on the initiation of breastfeeding, milk reserves, the attitude of new-borns to breastfeeding and length of breastfeeding compared to natural delivery. Studies have shown that a scheduled caesarean section is associated with early cessation of breastfeeding (20). Therefore, mothers who delivered by caesarean section may particularly need lactation counselling. Nevertheless, the education of future mothers about the impact of delivery by caesarean section on the mother's and child's health and proper breastfeeding is not sufficient and should be expanded (20).

We found that maternal education significantly affects the duration of breastfeeding (*p* = 0.0167). Over half of the participants were highly educated. Women who were breastfeeding for 6 months and longer were characterised by a higher education level (9). Studies confirm that the level of education significantly influences the length of breastfeeding. The average duration of breastfeeding increases with the level of mother's and father's education (1, 11, 12, 14). According to the Central Statistical Office (Główny Urząd Statystyczny), significantly more people aged 18–69 years undertake education in cities compared with villages (21). Therefore, the limited access to education and health care and worse financial situation of rural residents may have a negative impact on breastfeeding duration (22). Special educational programmes and campaigns aimed at people who live in villages would raise awareness and extend knowledge of breastfeeding benefits.

In this study, different durations of breastfeeding were observed depending on the region of Poland. Studies conducted among infants and children confirmed the occurrence of differences in frequency and duration of breastfeeding in different parts of Poland (23,24,25). In Central and Eastern Poland, 68.6% of infants were fed with breast milk for 6 months of age and only 3.7% exclusively (24). Only one-fourth of infants in the Kuyavian-Pomeranian Voivodeship up to 4 months of age were fed with breast milk exclusively and only 14% up to 6 months of age (23). Despite the awareness of the problem and the advantages of breastfeeding for both the baby and mother, research unchangeably confirms the insufficient duration of natural feeding in Poland (10, 26). Carrying out more specific studies in various regions of Poland would allow appropriate and more approachable programmes to be implemented for the promotion of breastfeeding depending on the needs in particular regions and different factors that influence breastfeeding duration.

Our findings showed that birth weight is a significant factor influencing breastfeeding. Flaherman et al. confirmed that postnatal weight loss and maternal fear, which make breastfeeding ineffective, have been reported in the literature as a reason for breastfeeding termination. According to the authors, reassuring mothers about normal newborn weight trends may reduce the risk of breastfeeding cessation (27). Furthermore, abnormal birth weight (<2,500 g or >4,000 g) may be associated with the increased risk of obesity in childhood as well as in adulthood. Knowing that breastfeeding decreases the risk of obesity, there is a need for better education about the benefits of breastfeeding and the influence of metabolic programming (8, 28).

In this study, the main reason for breastfeeding cessation was maternal concerns about insufficient milk supply. The study confirmed that the most frequently indicated reasons for cessation of breastfeeding at 2 months after a child's birth were nursing discomfort, insufficient milk and the concern that the breast milk alone did not meet the baby's needs. In addition, with the growing age of the child, self-weaning reasons were cited as important causes for stopping breastfeeding (29). Therefore, there is a need to establish a friendly environment for women who struggle with breastfeeding difficulties. Wider access to a lactation adviser, a dietitian and a midwife should be guaranteed. Women cannot be left alone when they notice difficulties with breastfeeding.

The strong points of our study are the large nationwide sample size of breastfeeding women and well-defined inclusion criteria which allow estimating the percentage of women who were exclusively breastfeeding in compliance with the WHO's recommendations. The study's limitations include the fact that the maximum duration of breastfeeding was not precisely stated, and the available data included censored observations. Some children might have been breastfed at the time of the study. Moreover, there is no information about the age of children at the time of the study. In several studies, the authors used a survival analysis such as a life-table technique, which is a valuable method to evaluate breastfeeding in due time (11, 30).

## Conclusion

In conclusion, despite many educational programmes, which are aimed at future parents and at improving knowledge on the benefits of human milk, breastfeeding duration rates are still not satisfying. Maternal age and BMI were found to be the factors significantly influencing exclusive breastfeeding duration and the most frequently cited reasons for breastfeeding cessation were maternal concerns about insufficient milk supply. Our study highlights that the reasons for breastfeeding cessation are often complex. Therefore, the promotion of breastfeeding for the first 6 months of life should be a social responsibility.
